# Tissue-type plasminogen activator-primed human iPSC-derived neural progenitor cells promote motor recovery after severe spinal cord injury

**DOI:** 10.1038/s41598-019-55132-8

**Published:** 2019-12-17

**Authors:** Yasuhiro Shiga, Akina Shiga, Pinar Mesci, HyoJun Kwon, Coralie Brifault, John H. Kim, Jacob J. Jeziorski, Chanond Nasamran, Seiji Ohtori, Alysson R. Muotri, Steven L. Gonias, Wendy M. Campana

**Affiliations:** 1 Department of Anesthesiology, University of California San Diego, La Jolla, CA 92093 USA; 2 Department of Orthopaedic Surgery and Graduate School of Medicine, Chiba University, Chiba, 260-8670 Japan; 3 Departments of Pediatrics and Cellular and Molecular Medicine, and the Stem Cell Program, University of California, San Diego, CA 92037-0695 USA; 4 Department of Pathology, University of California San Diego, La Jolla, CA 92093 USA; 5 Department of Chemistry, University of California, San Diego, La Jolla, CA 92093 USA; 6 Center for Computational Biology & Bioinformatics (CCBB), University of California, San Diego, La Jolla, CA 92093 USA; 7 Veterans Administration San Diego HealthCare System, San Diego, CA 92161 USA

**Keywords:** Spinal cord injury, Neural stem cells, Spinal cord diseases

## Abstract

The goal of stem cell therapy for spinal cord injury (SCI) is to restore motor function without exacerbating pain. Induced pluripotent stem cells (iPSC) may be administered by autologous transplantation, avoiding immunologic challenges. Identifying strategies to optimize iPSC-derived neural progenitor cells (*hi*NPC) for cell transplantation is an important objective. Herein, we report a method that takes advantage of the growth factor-like and anti-inflammatory activities of the fibrinolysis protease, tissue plasminogen activator tPA, without effects on hemostasis. We demonstrate that conditioning *hi*NPC with enzymatically-inactive tissue-type plasminogen activator (EI-tPA), prior to grafting into a T3 lesion site in a clinically relevant severe SCI model, significantly improves motor outcomes. EI-tPA-primed *hi*NPC grafted into lesion sites survived, differentiated, acquired markers of motor neuron maturation, and extended βIII-tubulin-positive axons several spinal segments below the lesion. Importantly, only SCI rats that received EI-tPA primed *hi*NPC demonstrated significantly improved motor function, without exacerbating pain. When *hi*NPC were treated with EI-tPA in culture, NMDA-R-dependent cell signaling was initiated, expression of genes associated with stemness (Nestin, Sox2) was regulated, and thrombin-induced cell death was prevented. EI-tPA emerges as a novel agent capable of improving the efficacy of stem cell therapy in SCI.

## Introduction

Stem cell therapy may be effective for treating patients with spinal cord injury (SCI). Substantial work has been conducted with embryonic neural stem cells (NSC) in animal models of SCI^1–3^. An alternative approach is to graft induced pluripotent stem cells (iPSC), which are pre-conditioned to generate lineage committed neural progenitor stem cells (NPC)^4–6^. iPSC are derived from skin^5^, dental pulp^6–8^, or blood cells^9^ and may be re-administered to patients by autologous transplantation. Grafting human iPSC-derived NPC (*hi*NPC) in patients with SCI avoids immunological and ethical complications associated with embryonic NSC.

*hi*NPC have shown efficacy in restoring motor function in rodent models of moderate lower thoracic (T9–T10) SCI^10–12^. In a severe cervical rodent model of SCI, grafted iPSC-derived NSC developed axons, which emerged from the implantation site and formed synapses with host spinal cord neurons; however, significant recovery of motor function was not observed^13^. It is not clear whether this result reflects the NSC phenotype^14^, insufficient optimization of the grafting method, or the severity of the SCI model. Accordingly, more studies are needed to understand *hi*NPC biology and to improve methodologies for successful *hi*NPC transplantation. The goal of this research project was to identify strategies for optimizing the efficacy of *hi*NPC as a treatment for severe SCI.

Tissue-type plasminogen activator (tPA) is an activator of fibrinolysis and globally approved drug for treating non-hemorrhagic stroke^15^. The activity of tPA in fibrinolysis and stroke is based on its function as a protease^16^; however, recombinant tPA also interacts with cellular receptors such as the N-methyl-D-aspartate Receptor (NMDA-R)^17,18^ and LDL Receptor-related Protein-1 (LRP1)^19^ to mediate changes in cell physiology that are independent of its protease activity and potentially relevant to the challenges of stem cell therapy. tPA is neuroprotective towards cortical neurons^20^ and promotes neurite outgrowth in neurons and neuron-like cells by activating cell-signaling factors such as c-Src and ERK1/2^21,22^. tPA also may regulate innate immunity by suppressing Toll-like Receptor responses^23^. These activities are replicated by enzymatically-inactive (EI) tPA.

In the normal CNS, tPA is present in pre-synaptic vesicles^24^ and secreted by neuronal growth cones, where it functions to promote synaptic plasticity, axonal elongation, and path-finding^25^. tPA expression is increased in Purkinje neurons during motor task training and has been implicated in activity-based cerebellar motor learning^26^. Collectively, these studies demonstrate that in addition to its role in fibrinolysis, tPA functions like a growth factor, eliciting receptor-mediated effects on cell-signaling and gene expression in various cells including motor neurons and neuron-like cells. The activity of tPA in iPSC and *hi*NPC has not been examined.

In this study, we tested whether the growth factor-like activities of EI-tPA may be exploited to improve the efficacy of transplanted iPSC-derived *hi*NPC in severe SCI in rats. We used a severe T3 SCI model (T3SCI), which induces motor loss and neuropathic pain^27^ and thus, models SCI in patients^28^. Our results demonstrate significant improvement in motor function in rats grafted with *hi*NPC, only when these cells are primed by treatment with EI-tPA. Cell fate mapping confirmed axon extension and differentiation of EI-tPA-treated *hi*NPC into motor neurons. EI-tPA induced cell-signaling in *hi*NPC *in vitro* and regulated expression of genes in *hi*NPC, including Nestin and Sox2. We conclude that EI-tPA primes *hi*NPC and improves functional regeneration in SCI. Because these activities are elicited with the EI derivative of tPA, abnormalities in hemostasis are not a concern.

## Results

### *hi*NPC respond to EI-tPA *in vitro*

**Figure 1:**
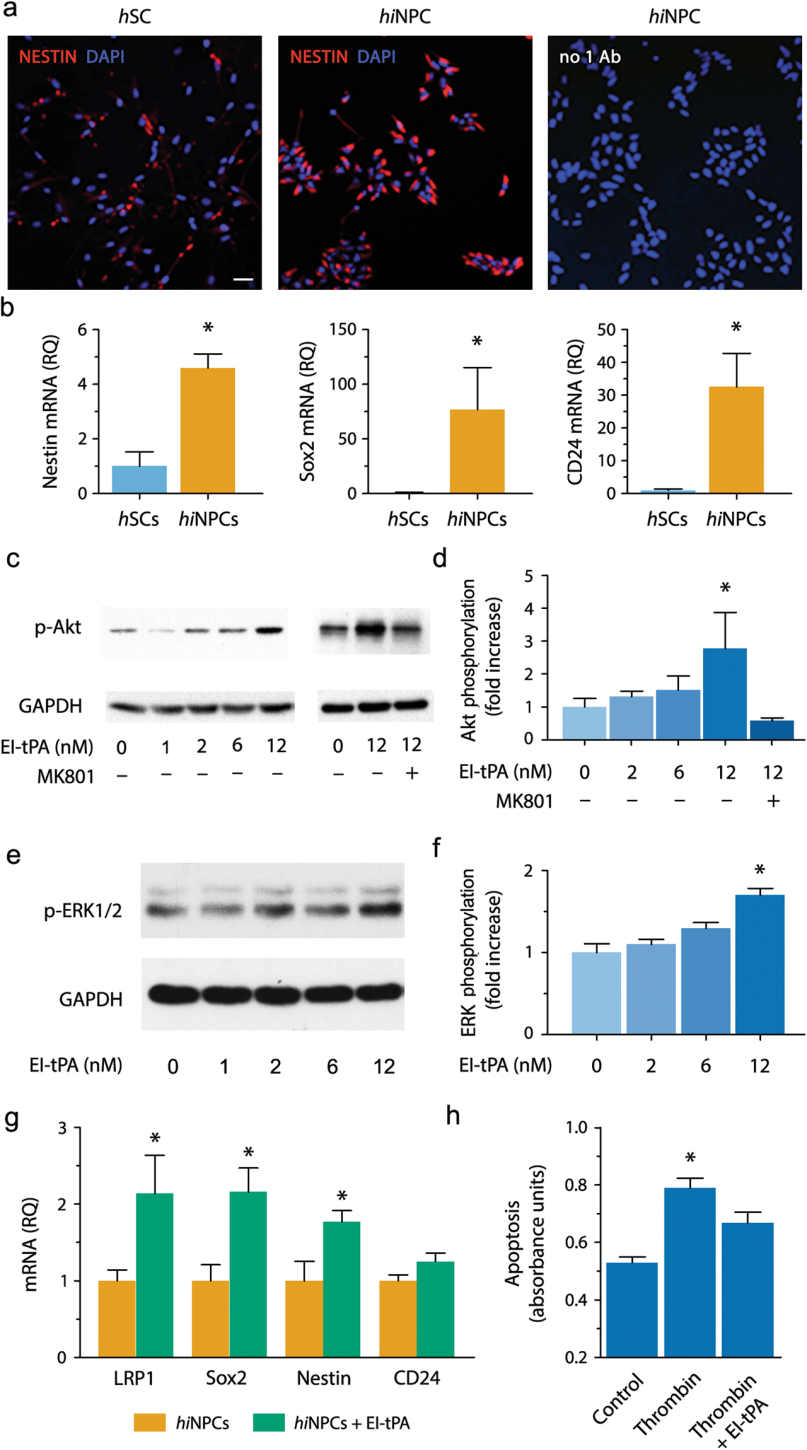
Bioactivity of EI-tPA in *hi*NPC *in vitro*. (**a**) Representative IF microscopy images of *hi*NPCs and *h*SCs immunostained to detect Nestin (red) and labeled with DAPI (blue) (*n* = 3 per group). As a control, *hi*NPC were incubated with secondary antibody only and with DAPI (right-hand panel). Scale bar 40 µm. (**b**) mRNAs encoding Nestin, Sox2, and CD24 in *hi*NPC and *h*SCs. Human EID2 mRNA was quantified as a normalizer (*P < 0.05 by Student's *t*-test, mean ± SEM; *n* = 4). (**c**) *hi*NPC were treated with EI-tPA (0–12 nM) for 15 min (*n* = 3 indepe*n*dent studies). In some wells, MK801 (1 µM) was added prior to EI-tPA (*n* = 2 independent studies). Equal amounts of cellular protein (20 µg) were subjected to immunoblot analysis to detect phospho-Akt and GAPDH. (**d)** Akt phosphorylation immunoblots were subjected to densitometry. Levels of phospho-Akt were standardized using GAPDH (*P < 0.05 by one-way ANOVA, Neuman Keuls *post hoc* test, mean ± SEM). (**e**) *hi*NPC in culture were treated with EI-tPA (0–12 nM). ERK1/2 phosphorylation and total ERK1/2 were determined. (**f**) Densitometry analysis of phospho-ERK1/2 and GAPDH (*P < 0.05 by one-way ANOVA followed by Neuman Keuls *post hoc* test, mean ± SEM; *n* = 3 independent experiments). (**g**) Cultured *hi*NPC were treated El-tPA (12 nM) or vehicle for 48 h. RT-qPCR was performed to compare mRNA levels for Nestin, Sox2, CD24, and LRP1 (*P < 0.05 by Student *t*-test, mean ± SEM; *n* = 5 replicates per group;). (**h**) *hi*NPC were treated with thrombin (5 units/mL), in the presence of vehicle or 12 nM EI-tPA for 18 hours. Cell extracts were analyzed using the Cell Death ELISA (*P < 0.05 by one-way ANOVA with a Neuman Keuls *post-hoc* test, mean ± SEM; *n* = 3–8 replicates per group).

We characterized *hi*NPC, which are iPSC-derived, lineage committed NPC^5,6^. Immunofluorescence analysis demonstrated that *hi*NPC (C6WT183) in culture express the stemness biomarker, Nestin, to a greater extent than human Schwann cells (*h*SC), which were analyzed as a control (Fig. 1a). RT-qPCR confirmed that the mRNAs encoding Nestin and Sox2 are greatly enriched in *hi*NPC compared with *h*SC (Fig. 1b). The pre-neuronal marker, CD24, also was abundantly expressed by *hi*NPC. Thus, *hi*NPC expressed stem cell and pre-neuronal markers prior to grafting, as anticipated.

Next, we examined whether EI-tPA elicits cell-signaling in cultured *hi*NPC. *hi*NPC were serum-starved and subsequently treated with EI-tPA (0–12 nM). In *hi*NPC treated with 12 nM EI-tPA, the survival-promoting cell-signaling factor, Akt (M.W.60 kDa), was phosphorylated within 15 min (Fig. 1c). MK-801 blocked Akt phosphorylation by EI-tPA, suggesting an essential role for the NMDA-R^19,21,23^. Densitometry analysis of five separate experiments demonstrated that the increase in Akt phosphorylation induced by EI-tPA and the effects of MK-801 were statistically significant (*P* < 0.05; Fig. 1d). EI-tPA (12 nM) also activated ERK1/2 (M.W. 42 and 44 kDa) in *hi*NPCs (*P* < 0.05; Fig. 1e,f).

We also identified the effects of EI-tPA on *hi*NPC gene expression. *hi*NPC were treated with 12 nM EI-tPA for 48 h. Expression of the stemness biomarkers, Nestin and Sox2, was significantly increased (Fig. 1g). Expression of LRP1, which serves as a co-receptor in EI-tPA-induced NMDA-R-dependent cell-signaling^19,22^, also was increased, whereas CD24 was unchanged. In trypan blue exclusion assays, thrombin, a known inducer of neuronal cell death^29,30^, dose-dependently decreased *hi*NPC viability (Fig. S1a). EI-tPA (12 nM) blocked cell death mediated by thrombin (5 units/mL) in cultured *hi*NPC (*P* < 0.05; Fig. S1b). Cell Death Elisa™ studies confirmed that the viability of *hi*NPC was decreased by thrombin and rescued by EI-tPA (*P* < 0.05 Fig. 1h). Increased expression of Sox2 provides one explanation for improved resistance of EI-tPA-treated *hi*NPC to apoptosis^31^.

To confirm that our results with EI-tPA and *hi*NPCs are representative of *hi*NPCs in general, we tested a second iPSC-derived *hi*NPC line from a different patient (C5WT126). A similar characterization pattern, including expression of the stem cell biomarkers, Nestin, Pax6 and Brn2, was observed in both the first and second *hi*NPC clones (Fig. S2). When the second clone was treated with 12 nM EI-tPA, Akt was phosphorylated within 15 min and this response was blocked by MK801 (1.0 μM) (Fig. S3). Glycogen synthase kinase 3β (GSK-3β) was phosphorylated in response to EI-tPA, as was ERK1/2. These phosphorylation events also were inhibited by MK-801 (Fig. S3), confirming an essential role for the NMDA-R^19,21,23^, as anticipated^17,18,23^.

### *hi*NPC improve locomotor activity in severe SCI only when pre-treated with EI-tPA

Next, we suspended *hi*NPC in fibrin and grafted the cells into immunodeficient rats one week following T3 spinal cord compression injury (T3SCI). Histologically, T3SCI generates a band of disrupted parenchyma across the compression site with partial cavitation^27^. Thirteen rats were grafted with GFP-expressing *hi*NPC that were pre-treated with EI-tPA (12 nM) for 15 min. Eleven rats were grafted with GFP-expressing *hi*NPC that were not treated with EI-tPA. Eleven additional control rats were grafted with cell-free vehicle. All grafts contained a lower concentration of thrombin, compared with that previously used^13^ given the potential for thrombin to cause cell stress and cell death (Fig. 1h). The grafting timepoint was selected for clinical relevance to SCI patients undergoing decompression and stabilization^32^.

**Figure 2:**
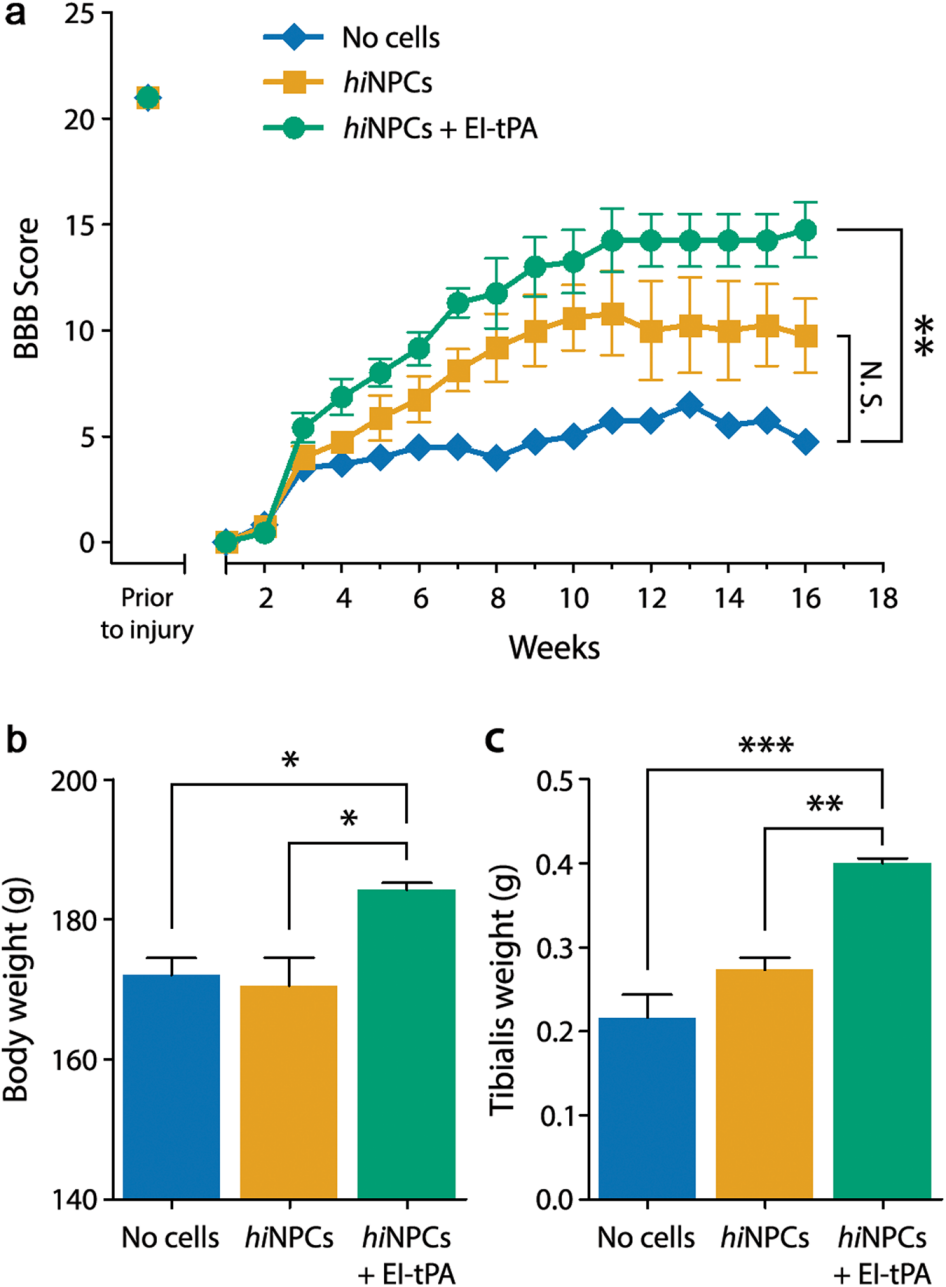
*hi*NPC primed with EI-tPA enhance motor recovery in T3SCI after four months. (**a**) BBB scores in rats grafted with *hi*NPC (yellow) or EI-tPA-treated *hi*NPC (green) or vehicle (no cell control) (blue) after T3SCI (**P < 0.01 by repeated measures ANOVA followed by Bonferroni *post-hoc* test, mean ± S.E.M; *n* = 14 animals). (**b**) Body weights in rats grafted with vehicle (no cell control) (blue), *hi*NPC (yellow), or EI-tPA-treated *hi*NPC (green) after T3SCI (*P < 0.05 by one-way ANOVA and Tukey's *post-hoc* test, mean ± S.E.M; *n* = 18 animals). (**c**) Tibialis anterior muscle weight in rats grafted with vehicle (no cell control) (blue), *hi*NPC (yellow) or EI-tPA-treated *hi*NPC (green) after T3SCI (**P < 0.01; ***P < 0.001 by one-way ANOVA and Tukey's *post-hoc* test, mean ± S.E.M; *n* = 18 animals).

Rats subjected to T3SCI had complete hindlimb paralysis within a day after injury. T3SCI caused substantial loss of motor function, when assessed two weeks post-injury, as determined using the Basso, Beattie and Bresnahan (BBB) locomotor scale (Fig. 2a)^33^. BBB scores were <2. By 3 weeks, rats without grafted *hi*NPC recovered to a BBB score of 4 but failed to improve beyond a BBB score of 5 for the duration of the study (4 months), consistent with previous results^27^.

Rats that were subjected to T3SCI and grafted with *hi*NPC demonstrated a trend towards improved BBB scores beginning at 3–6 weeks; however, throughout the 16-week observation period, statistical significance was not achieved. In rats grafted with EI-tPA-treated *hi*NPC, BBB scores were further improved and the effects of EI-tPA-treated *hi*NPC on motor function achieved statistical significance. BBB scores of approximately 15 were recorded at 16 weeks (*P* < 0.01; Fig. 2a). Representative video imaging of rats subjected to T3SCI (Movie S1) and grafted with EI-tPA-treated *hi*NPC demonstrated obvious improvements compared with rats that were grafted with untreated *hi*NPC (Movies S2, S3). The significant effects of EI-tPA on the efficacy of grafted *hi*NPC were confirmed by analyzing total body weight (Fig. 2b) and tibialis anterior leg muscle weight (Fig. 2c), parameters known to correlate with motor recovery following SCI^34^.

### *hi*NPC survive and differentiate in injured spinal cords

**Figure 3:**
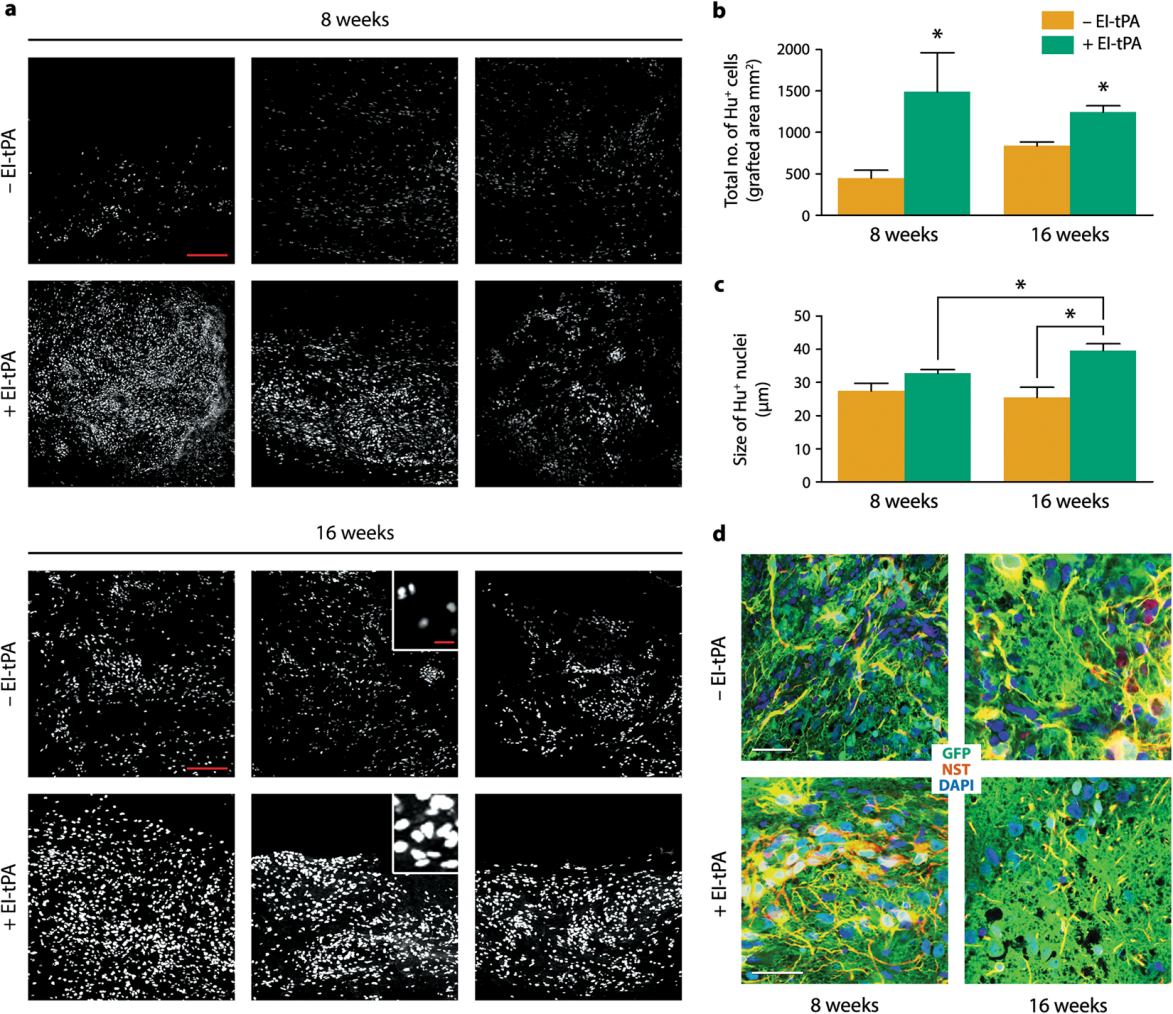
*hi*NPC survival and maturation *in vivo* are enhanced by EI-tPA. (**a**) Fluorescent images of the human nucleus marker, hNu^+^ (white), in T3SCI lesion sites with *hi*NPC and EI-tPA-treated *hi*NPC at 8 and 16 weeks. Each panel represents an individual rat. Scale bars for low power (200X) images in (**a**) are 200 µm and for insets are 25 µm. (**b**) Quantification of hNu^+^ cells in lesions with *hi*NPC (yellow) and EI-tPA-treated *hi*NPC (green) at 8 and 16 weeks (*P < 0.05 by Student's *t*-test, mean ± SEM; *n* = 3 per group). (**c**) Quantification of the size of hNu^+^ nuclei of EI-tPA-treated *hi*NPC versus untreated *hi*NPC (*P < 0.05 by Student's *t*-test, mean ± SEM; *n* = 3 per group). (**d**) Dual label immunofluorescence of GFP in *hi*NPCs (green) and the stem cell marker, Nestin (red), shows co-localization in grafted *hi*NPC. Nuclei are labeled with DAPI (blue). Scale bars for images (400X) are 50 µm.

Grafted *hi*NPC survived and filled the SCI lesion cavity. Immunostaining for hNu, which specifically detects human nuclei, showed a three-fold increase in the number of *hi*NPC in the EI-tPA-treated group (*P* < 0.05) 8 weeks after T3SCI, suggesting greater survival and/or *hi*NPC proliferation (Fig. 3a,b). At 16 weeks, the number of grafted *hi*NPC in the EI-tPA-treated group remained two-fold higher (*P* < 0.05; Fig. 3b). Importantly, the mean size of the hNu^+^ nuclei was significantly increased in grafts of EI-tPA-treated *hi*NPC, compared with animals that received untreated *hi*NPC (~40 µm vs. ~25 µm) (*P* < 0.05; Fig. 3a,c). Nuclear hypertrophy is an indicator of cell maturation^3^.

Nestin is a nucleo-cytoplasmic shuttling protein that may serve as an indicator of *hi*NPC differentiation^35^. At 8 weeks, Nestin immunoreactivity was most intense in the peri-nuclear area of GFP-expressing EI-tPA-treated *hi*NPC, however, untreated *hi*NPC demonstrated Nestin immunoreactivity only in cytoplasmic processes (Figs. 3d and S4). At 16 weeks, perinuclear nestin immunoreactivity was observed in *hi*NPC that were not EI-tPA-treated. Thus, *hi*NPC may have undergone a similar transition in nestin subcellular localization irrespective of whether EI-tPA is present. EI-tPA may have accelerated the transition.

**Figure 4:**
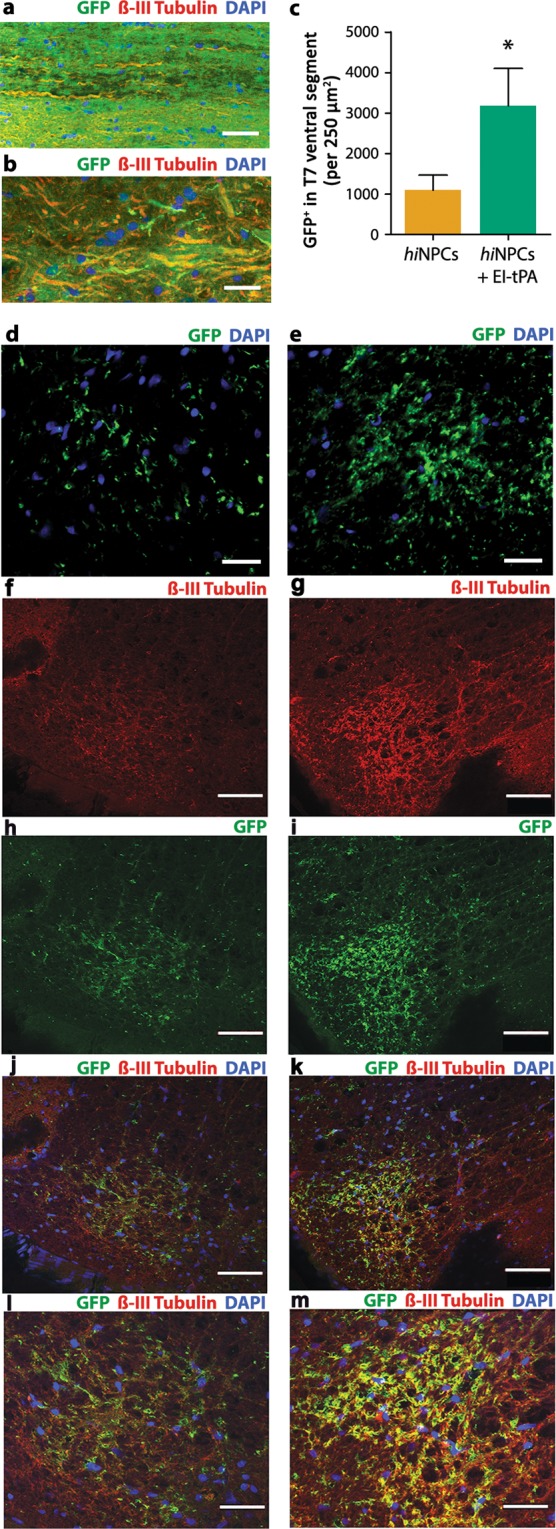
EI-tPA promotes penetration of *hi*NPC axons into the ventral spinal T7 segment *in vivo*. (**a**) GFP-expressing EI-tPA-treated *hi*NPC co-localized with axonal marker, βIII tubulin (Tuji) emerging from the grafting site after 4 months. DAPI labels nuclei (blue). Images represent 3 individual rats per group. Scale bar is 50 µm. (**b**) Representative higher magnification image of (**a**). Scale bar is 25 µm. (**c**) Quantification of GFP immunoreactivity in the host ventral T7 segment in rats grafted with EI-tPA-treated *hi*NPC versus untreated *hi*NPC (*P < 0.05 by Students *t*-test, mean ± S.E.M; *n* = 3 rats per group). Fluorescent images of graft derived GFP^+^ cells integrating into T7 spinal segments; (**d**) *hi*NPC or (**e**) EI-tPA tr**e**ated *hi*NPC. Scale bar is 30 µm. Dual labeling immunofluorescence of βIII tubulin and GFP in T7 ventral spinal segments; (**f,h,j,l)** *hi*NPC or (**g,i,k,m**) EI-tPA treated *hi*NPC. Scale bar 50 µm.

To determine whether *hi*NPCs extend and integrate into the host spinal cord, we examined longitudinal sections beginning at the injury site and extending caudally βIII tubulin-positive axons emerged from the lesion site and co-localized with GFP immunoreactivity in rats grafted with untreated *hi*NPCs or EI-tPA-treated *hi*NPCs (Fig. 4a,b). Next, we compared transverse T7 sections isolated from rats grafted with untreated *hi*NPCs or EI-tPA-treated *hi*NPCs to determine whether EI-tPA promoted axonal extension. Although GFP immunopositivity was detected in the white and gray matter in both experimental groups, in rats grafted with EI-tPA-treated *hi*NPCs, GFP immunoreactivity appeared substantially increased in the T7 spinal segment (dorsal and ventral areas) (Fig. 4d,e). Quantification of total GFP-immunopositive tissue in the T7 ventral segment showed a 3-fold increase in rats grafted with EI-tPA-treated *hi*NPCs (*P* < 0.05; Fig. 4c). Given the size of the immunoreactive projections (<2 µM) and lack of co-localization of GFP with DAPI, the GFP most likely represents *hi*NPC-derived axons and not migrating glia.

To confirm that *hi*NPC expressed a neuronal phenotype, we performed dual-label immunofluorescence for GFP and βIII tubulin-positive projections in T7 transverse sections. SCI rats treated with *hi*NPCs in the absence of EI-tPA expressed some βIII tubulin (Fig. 4f) and GFP immunoreactivity (Fig. 4h) in the spinal ventral horn, however, minimal colocalization was apparent (Fig. 4j,l). By contrast, SCI rats grafted with EI-tPA-treated *hi*NPC robustly expressed βIII tubulin (Fig. 4g) and GFP immunoreactivity (Fig. 4i) in the spinal ventral horn. The majority of GFP co-localized with βIII tubulin (Fig. 4k,m). These finding indicate that EI-tPA-treated *hi*NPC grafted into the lesion site extend neuronal projections four segments distal to the lesion site after 4 months.

**Figure 5:**
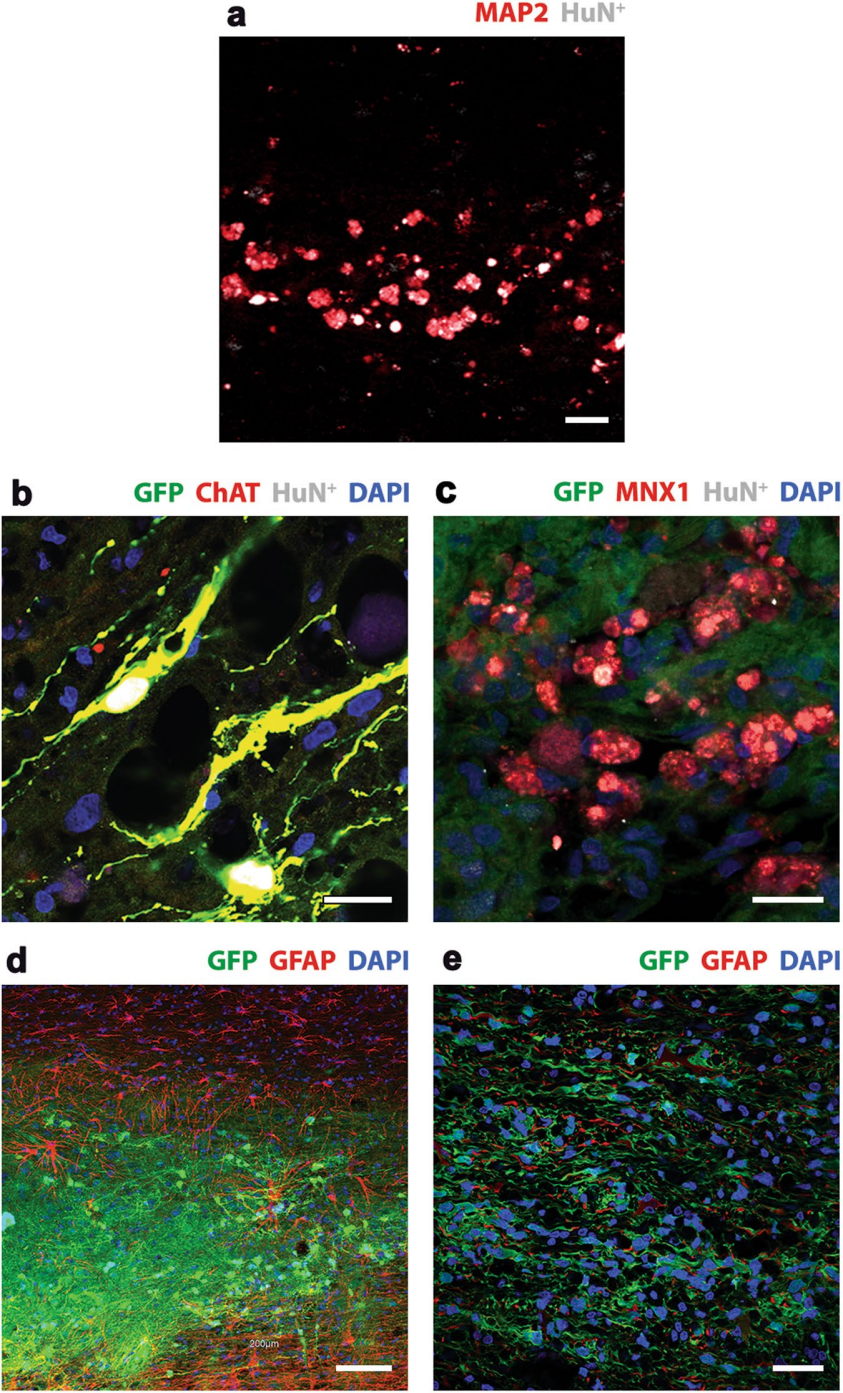
Cell fate mapping of EI-tPA treated *hi*NPC *in vivo*. (**a**) Dual label immunofluorescence showed co-localization with hNu (white) and the neuron-specific marker, MAP2 (red), at the lesion site. Scale bar 25 µm. (**b**) Triple label immunofluorescence showed co-localization with ChAT (red), hNu (white) and GFP (green), revealing the presence of mature motor neurons at the lesion site by 8 weeks. Scale bar is 25 µm. (**c**) Triple label immunofluorescence showed co-localization of Mnx1 (red), hNu (white) and GFP (green), revealing the presence of mature motor neurons at the lesion site at 16 weeks. DAPI labels nuclei (blue). Scale bar is 25 µm. (**d,e**) Immunofluorescence showing GFP expressing *hi*NPC in the lesion site (low power) that is distinct from GFAP immunoreactive host astrocytes (red) at 8 weeks. Scale bar is 100 µm. (**f**) Higher power image distal to the lesion site. GFP expressing *hi*NPC were integrated into the host tissue and did not co-localize with GFAP (red) at 16 weeks. Scale bar 100 µm.

Cell fate mapping studies at the lesion site revealed that *hi*NPC acquire a neuron phenotype. *hi*NPC identified by hNu+ (as shown in Fig. 3a) co-localized with the neuron specific cytoskeletal protein MAP2 in lesion sites (Fig. 5a). This was observed in both *hi*NPC grafts alone and EI-tPA-treated *hi*NPC grafts. At 8 weeks, the motor neuron marker, choline acetyltransferase (ChAT), co-localized with GFP and hNu (Fig. 5b) in EI-tPA-treated *hi*NPCs. Motor Neuron and Pancreas Homeobox 1 (HBP/MNX1), a second motor neuron marker found in nuclei^36^, also co-localized with GFP and hNu (Fig. 5c). GFP-expressing, EI-tPA-treated *hi*NPC failed to co-localize with GFAP in the lesion site (Fig. 5d) and distal to the lesion site (Fig. 5e), suggesting that the *hi*NPC did not become astrocytes.

### Nociception in rats subjected to T3SCI and treated with *hi*NPC with and without EI-tPA

**Figure 6:**
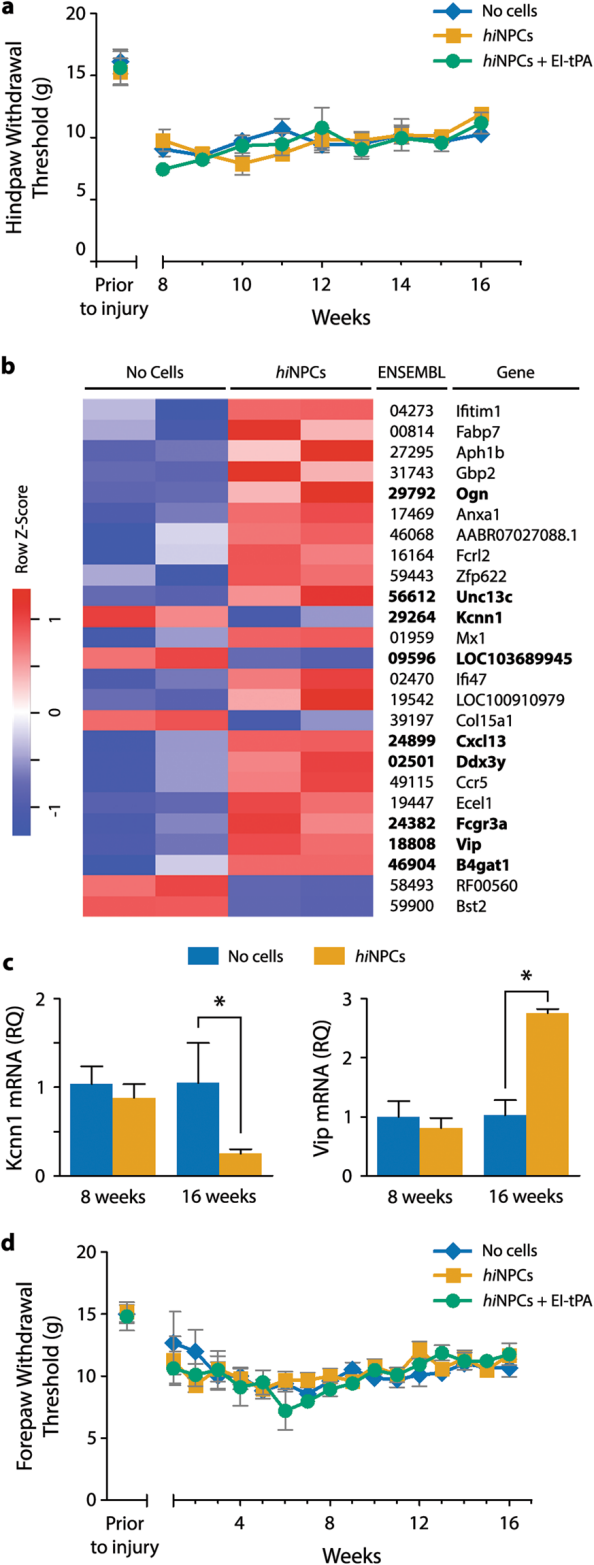
Effects of *hi*NPC on sensory function following T3SCI. (**a**) Hindpaw tactile allodynia was studied from 8 to 16 weeks post-T3SCI. (n.s., by two-way repeated measures ANOVA followed by Bonferroni *post-hoc* analysis mean ± SEM; *n* = 4–6 rats per group). (**b**) Heat map of differentially abundant transcripts by RNA-Seq in L5 DRGs after T3SCI, with and without grafted *hi*NPC. Increased expression is shown in red and reduced expression is shown in blue. RNA-Seq transcripts and experimental groups are arranged by unsupervised hierarchical clustering. Genes in bold are of potential interest for future pain related studies. (**c**) Changes in expression of KCNN1 and VIP in L5 DRGs at 8 and 16 weeks post-T3SCI (*P < 0.05 by Students *t*-test, mean ± SEM; *n* = 2–3). (**d**) Forepaw tactile withdrawal thresholds from 8–16 weeks (n.s., two-way repeated measures ANOVA followed by Bonferroni *post-hoc* analysis, mean ± SEM; *n* = 4–6 rats per group).

To test whether rats grafted with *hi*NPC develop altered sensation to light touch, which may indicate exacerbation of injury-associated neuropathic pain, we performed hindpaw tactile allodynia testing 8 weeks post-T3SCI, when motor improvement was underway. Prior to T3SCI, rats had a mean paw withdrawal threshold (PWT) of 15.7 ± 0.9 g (Fig. 6a). Eight weeks after T3SCI, the PWT was reduced to 8.1 ± 0.9 g in rats that received no *hi*NPC, indicating allodynia. Importantly, the extent and magnitude of allodynia was not altered by *hi*NPC or by EI-tPA-treated *hi*NPC through the duration of the study (8–16 weeks post-T3SCI).

To mine for changes in gene expression that might predict altered somatosensory function, we performed RNA-Seq analysis of DRGs 16 weeks post-T3SCI and grafting of *hi*NPCs. Control animals were subjected to T3SCI but not grafted. Differentially expressed (DE) genes were identified and expressed as logFC (Fig. 6b). Amongst the top 25 regulated genes, RNA-Seq identified the known pain-related genes, KCNN1 and VIP (Table S1). To validate KCNN1 and VIP as genes regulated in the DRG in response to *hi*NPC grafting at the SCI site, RT-qPCR was performed. At 8 weeks, expression of KCNN1 or VIP was not significantly different in the control and *hi*NPC-treated groups (Fig. 6c). However, 16 weeks post-SCI, KCNN1 mRNA was decreased in the *hi*NPC-treated group, whereas VIP mRNA was increased (*P* < 0.05; Fig. 6c). These changes in DRG gene expression suggest that *hi*NPC may, in the long term, induce changes in somatosensory function that were not detected in our tactile allodynia experiments.

Forepaw tactile allodynia was also studied since neuropathic pain may be observed above or at the level of injury^27^. Prior to T3SCI, control rats had a mean forepaw PWT of 15.1 ± 1.0 g (Fig. 5d). Four weeks after SCI, the PWT in control rats was 9.2 ± 0.8 g. Rats grafted with *hi*NPC or EI-tPA-treated *hi*NPC showed no significant differences in forepaw PWTs over the 16-week monitoring period, indicating that neuropathic pain was not exacerbated or mitigated by the *hi*NPC.

## Discussion

This study demonstrates that the efficacy of *hi*NPC in facilitating SCI repair and recovery of motor function may be significantly improved by treating the *hi*NPC with EI-tPA. Compared with untreated *hi*NPC, EI-tPA-treated *hi*NPC demonstrated improved survival and maturation at the SCI lesion site and a significant increase in the number of axons projecting caudally into spinal segment T7. Although a trend towards improved motor function was observed in animals grafted with untreated *hi*NPC, the magnitude and extent of the response to *hi*NPC grafting was statistically significant only when the *hi*NPC were EI-tPA-primed. To our knowledge, this is the first study showing beneficial effects of EI-tPA on neural progenitor cells and in particular, *hi*NPC, *in vitro* and *in vivo*.

In studies with cells in culture, we showed that EI-tPA activates cell-signaling cascades in *hi*NPC that are associated with cell survival and dependent on the NMDA-R. In addition to its role as an ionotropic glutamate receptor, the NMDA-R is a known cell-signaling receptor for tPA, which may function in conjunction with LRP1^17–19,22^. EI-tPA increased expression of LRP1 mRNA in *hi*NPC, which may support or amplify tPA-dependent cell signaling and cell survival. LRP1 is a potent cell survival receptor for Schwann cells^37^. Cooperation between LRP1 and the NMDA-R in EI-tPA-signaling in *hi*NPC suggests that this receptor system is conserved in diverse cell types.

When *hi*NPC were challenged with thrombin *in vitro*, an inducer of cell death in neurons^29,30^ and a component of the SCI grafting cocktail, EI-tPA protected the *hi*NPC from cell death. EI-tPA-dependent cell signaling also increased expression of stem cell markers, Sox2 and Nestin. Accumulating evidence suggests that when stem cells express increased levels of the stemness factors, Sox2 and Oct4, they have a greater intrinsic resistance towards apoptosis^31^. Pre-treating *hi*NPC with EI-tPA prior to grafting may protect these cells from the challenging, pro-inflammatory SCI microenvironment. The hypothesis that EI-tPA may promote *hi*NPC survival *in vivo* in SCI is supported by the work of LeMarchand *et al*.^38^, demonstrating neuroprotective effects of tPA on stressed postnatal neurons. Nonetheless, the effects we observed in our studies with EI-tPA and *hi*NPC in culture is likely dictated by the *in vitro* niche and should be interpreted with this perspective.

Although previous studies demonstrated the ability of NSC to promote recovery after SCI^3,39^, the cells used in these studies were derived from embryonic tissues, which raise ethical issues when translation into human patients is considered. Although iPSC-derived NSC are previously reported to develop axons and form synapses when transplanted into SCI lesions, significant functional motor recovery was not observed^13^. We further confirmed this observation in our studies using *hi*NPC that were not treated with EI-tPA. Differences in tissue origin, induction methods, injury severity, the level of the SCI lesion, and grafting factors may contribute to variation in the results observed when iPSC technology is applied as a therapeutic strategy for SCI^4,10,11,13^. Notably, we utilized a well-described retroviral method to reprogram fibroblasts into *hi*PSC^4–6,40,41^. We previously used these methods in several studies and did not observe any aneuploidy or genomic errors due to the reprogramming method^4–6,42,43^. However, as we consider transplantation of stem cell grafts for SCI therapeutically, methods utilizing non-integrating reprogramming methods might be suitable for clinical translation^44^.

In addition to observing improved motor function in animals grafted with EI-tPA-treated *hi*NPC, we observed decreased muscle atrophy. ChAT and MNX1-positive neurons were present in lesions sites grafted with *hi*NPC which is consistent with functional motor improvements. It is possible that EI-tPA caused the *hi*NPC to differentiate into motor neurons at earlier time points. It is also possible that EI-tPA-treated *hi*NPC affected central pattern generators by activating key circuitry above the lesion site, which contributes to improved motor function^45,46^. Yet, the most striking result was the large number of axons penetrating into the T7 spinal ventral horn in the EI-tPA treated *hi*NPC group. Although there were GFP + structures in the T7 segment by the *hi*NPCs alone, they did not appear to be a neuronal phenotype, as they did not colocalize with an axonal marker, βIII tubulin. Whether this is due to the limited number and size of axonal projections or whether they differentiate into a glia phenotype remains unknown. However, the improvements we observed in motor function by EI-tPA priming can at least be partially explained by a greater density of axons. Importantly, host axons appeared to be more abundantly expressed, suggesting the EI-tPA primed *hi*NPCs may improve host axon regeneration. Further studies are needed to confirm these hypotheses.

Notably, *hi*NPC did not worsen or improve “above” or “below” the lesion site pain-related behaviors. In previous studies that reported detrimental effects of stem cells on pain, the grafted NSC expressed astrocyte markers *in vivo*^47^. Our model system differs markedly in that we graft *hi*NPC directly into the lesion site, generating numerous mature neurons that extend many axons into the host spinal cord. There was also a possibility that “below-level” pain could now become consciously detectable pain after *hi*NPC treatment since *hi*NPC appeared to re-establish motor neural connectivity. However, we observed no detectable alterations in hindlimb sensation in rats grafted with *hi*NPC or *hi*NPC with EI-tPA, despite the improved motor recovery.

Although we did not see any differences in functional pain outcomes amongst *hi*NPC groups even when primed with EI-tPA in SCI rats, we performed RNA sequencing (RNA-Seq) studies in DRGs to determine whether changes in the molecular pain processing signature reflect *hi*NPC treatment after 16 weeks. We observed differential gene expression in 11 potential pain related processing genes that included VIP and KCNN1. Increased VIP mRNA has been associated with neuropathic pain^48^ and KCNN1 down regulation has been implicated in pain hypersensitivity^49^. Importantly, these genes were unchanged in the DRGs after 8 weeks, but were significantly altered in rats grafted with *hi*NPC after 16 weeks. Changes in expression of these genes may justify future studies in which somatosensory testing is examined over longer periods of time following *hi*NPC grafting.

## Methods

### Neural progenitor cells (NPC) and human schwann cells (SC)

Human iPSC were obtained from skin fibroblasts from two healthy patients, WT126 (one clone, C5) and WT83 (two clones, C6,C9) as previously described^5,40–43^. The study protocol was approved by the University of California, San Diego (UCSD) and the Salk Institute Institutional Review Board (IRB)/Embryonic Stem Cell Oversight Committee (ESCRO) committees. Studies were carried out in accordance with these relevant guidelines and regulations. Briefly, subjects were recruited through the University of California, San Diego Autism Center of Excellence from a pool of volunteers formerly included in previous brain imaging studies. After a complete description of the study was provided, written informed consent was obtained from all adult subjects. Primary cultures of human SCs isolated from human spinal nerve were purchased from ScienCell Research Laboratories (#1700; Carlsbad, CA) and maintained in the manufacturer′s Schwann Cell Medium (#1701), as previously described^50^.

### RNA isolation and qPCR

Cells or tissue were homogenized in lysis buffer and total RNA was extracted using the NucleoSpin® RNA kit (Macherey-Nagel). RNA was reverse-transcribed using the iScript cDNA synthesis kit (Bio-Rad). qPCR was performed using TaqMan® gene expression products and an AB Step one Plus Real-Time PCR System (Applied Biosystems), as previously described^23^. The mRNAs analyzed for hiNPCs and hSCs included: Nestin (Hs04187831_g1), Sox2 (Hs04234836_s1), CD24 (Hs02379687_s1), LRP1 (Hs00233856_m1), the turquoise module potassium channel related gene (KCNN1) (Rn00570904_m1). The mRNAs analyzed for dorsal root ganglia (DRGs) included: and vasoactive intestinal polypeptide, VIP (Rn01430567_m1). The relative change in mRNA expression was calculated using the 2^ΔΔCt^ method and EID2 (Hs00541978_s1) and GAPDH (Rn01775763_g1) mRNA as internal normalizers for human cells and rat tissues respectively. EID2 is a stable reference gene for human iPSCs^51^. Control qPCR reactions were performed using samples that were not exposed to reverse transcriptase to verify the absence of genomic DNA contamination.

### Cell signaling studies

*hi*NPC were transferred to serum-free medium for up to 6 h and then treated with EI-tPA or vehicle. When indicated, *hi*NPC were pre-treated with MK801 (1 µM; Tocris Bioscience) for 30 min. Immunoblot analysis was performed as described previously^37,52^.

### Cell death studies

*hi*NPC were plated in 96 well plates and exposed to thrombin (5 units/mL) with and without EI-tPA (12 nM). Cell death was determined using Cell Death Detection ELISA^PLUS^ according to the manufacturer's instructions (Roche).

### Animals

Studies were performed using 38 adult female Foxn1^rnu^ rats (150–160 g; Charles River, Wilmington, MA). We chose female rats for these studies because their bladders are more readily emptied following severe SCI. Inclusion of female subjects in SCI and pain studies is well supported^53^. Pain prevalence rates and descriptions of pain do not differ between male and female patients with SCI^54,55^.

### Surgeries

Animals were housed individually with free access to food and water in a vivarium approved by the American Association for the Accreditation of Laboratory Animal Care (AAALC). All animal studies were carried out according to protocols approved by the Institutional Animal Care and Use Committee (IACUC) at the University of California, San Diego, CA and the Veterans Healthcare System following the International Association for the Study of Pain (IASP) Guidelines for Use of Animals in Research. Rats underwent surgery under deep anesthesia using isoflurane (5% initially and then 3% for maintenance during surgery). An incision was made in the skin of the back over the T2 spinous process. After clearing of the muscle, the dorsal aspect of the T3 vertebra was removed. The cord was compressed for 5 seconds using mosquito forceps locked completely closed. Thin forceps were used to bilaterally compress the cord at two sites in the center of the T3 spinal segment located 1 mm apart, as we described previously^27^. The muscles overlying the spinal cord were sutured and the skin incision was closed with surgical suture. Following surgery, rats were maintained in cages kept on heat pads (37 °C) for 1 week and received banamine (1 mg/kg) and ampicillin (0.2 mg/kg) in Ringer's lactate for three days. Bladder care was performed twice daily at 12-hour intervals for the first two weeks following surgery, and thereafter once daily until rats could urinate on their own, approximately four weeks after surgery. Bladders were always expressed prior to acclimation for behavioral testing. Rats were given amoxicillin their drinking water over the duration of the experiment to prevent bladder infections that could confound behavioral results.

### SCI and grafting surgeries

Grafting human iPSC derived neural progenitor cells into rodent models of spinal cord injury was approved by the IACUC at UCSD. Rats were subjected to T3SCI as previously described^27^. We used T3SCI because this is the highest spinal level at which a severe spinal cord lesion allows subject survival; severe lesions located more rostral than T3 result in persistent forelimb dysfunction. This procedure is a modification of the clip-compression model^56^. Two rats died after surgery. The remaining 36 rats were randomly divided into cohorts that received no cells with growth factor cocktail (n = 13), *hiNPC* (n = 11) or EI-tPA-treated *hiNPC* (n = 12). *hi*NPC were reconstituted in human fibrinogen (25 mg/ml, Sigma, F3879), and a cocktail of four growth factors: brain-derived neurotrophic factor (BDNF; 50 µg/ml, Peprotech, 452–02), basic fibroblast growth factor (bFGF; 10 μg/ml, Sigma, F0291), vascular endothelial growth factor, (VEGF, 10 µg/mL Peprotech 100–20), and a calpain inhibitor (MDL28170; 50 µM Sigma M6690) with or without EI-tPA (12 nM; Molecular Innovations). Immediately prior to injection into lesion sites, cells, fibrinogen and growth factor cocktails were mixed with rat thrombin (5U/ml, Sigma, T5772). Two injections were made into the lesion cavity. 1.5 million *hi*NPC*s* were grafted. Injections were stopped if reflux occurred. Treatments were initiated one week after T3SCI because at that time, acute inflammation is reduced and initiation of gliosis is minimized^57^.

### RNASeq studies

Total RNA isolated from DRGs of rats grafted with *hi*NPC or from control rats that did not receive *hi*NPC were analyzed. The RNA was subjected to RNASeq using Illumina High Seq. Quality control of the raw fastq files was performed using the software tool FastQC. Sequencing reads were aligned to the Ensembl rat genome (Rnor_6.0) using the STAR v2.5.1a aligner^58^. Read quantification was performed with RSEM^49^ v1.3.0 and Ensembl annotation (Rattus_norvegicus.Rnor_6.0.92.gtf). The R BioConductor packages edgeR^59^ and limma^60^ were used to implement the limma-voom^61^ method for differential expression analysis. The experimental design was modeled upon condition (~0 + condition). Contrasts were made between *hi*NPC and “no cell” groups.

### Statistics

Statistical analysis was performed using GraphPad Prism 5.0 (GraphPad Software Inc.). All results are expressed as mean ± SEM. Differences between two treatment means were assessed by a Student's t-test. Differences between multiple treatment means were analyzed by a one-way ANOVA followed by Neuman Keuls or Tukey’s *post hoc* tests. Behavioral data including BBB and tactile allodynia assessments were analyzed by repeated measures ANOVA followed by Bonferroni’s *post hoc* test. *P* < 0.05 was considered statistically significant.

## Supplementary information

Supplementary Information

Movie S1

Movie S2

Movie S3

## Data Availability

For additional methods on *hi*NPC, immunoblotting, trypan blue exclusions studies, motor and sensory functional testing, and immunofluorescence microscopy please see Supplemental Experimental Procedures. The data sets generated during and/or analyzed during the current study are available from the corresponding author on reasonable request.
