# Supplementary material for: Tissue-type plasminogen activator-primed human iPSC-derived neural progenitor cells promote motor recovery after severe spinal cord injury
Source: Sci Rep. 2019 Dec 17;9:19291. doi: 10.1038/s41598-019-55132-8 (PMC6917728; doi:10.1038/s41598-019-55132-8)
Supplement: Supplementary file 1 — Supplementary Information [file 41598_2019_55132_MOESM1_ESM.pdf]

Supplementary Information

Tissue-type plasminogen activator-primed human iPSC-derived neural progenitor cells promote motor recovery after severe spinal cord injury

Authors

Yasuhiro Shiga[a,b], Akina Shiga[b], Pinar Mesci[c], HyoJun Kwon[a], Coralie Brifault[a,d], John H. Kim[a,e], Jacob J. Jeziorski[c], Chanond Nasamran[f], Seiji Ohtori[b], Alysson R. Muotri[c], Steven L. Gonias[d], and Wendy M. Campana[a,g]*

[a]Department of Anesthesiology, University of California San Diego, La Jolla, CA, 92093, USA; [b]Department of Orthopaedic Surgery and Graduate School of Medicine, Chiba University, Chiba 260-8670, Japan; [c]Departments of Pediatrics and Cellular and Molecular Medicine, and the Stem Cell Program, University of California San Diego CA 92037-0695, USA; [d]Department of Pathology, University of California San Diego, La Jolla, CA, 92093, USA; [e]Department of Chemistry, University of California, San Diego, La Jolla, CA 92093, USA; [f]Center for Computational Biology & Bioinformatics (CCBB), University of California, San Diego, La Jolla, CA, 92093, USA; and [g]Veterans Administration San Diego HealthCare System, San Diego, CA, 92161, USA

*To whom correspondence should be addressed: email: wcampana@ucsd.edu

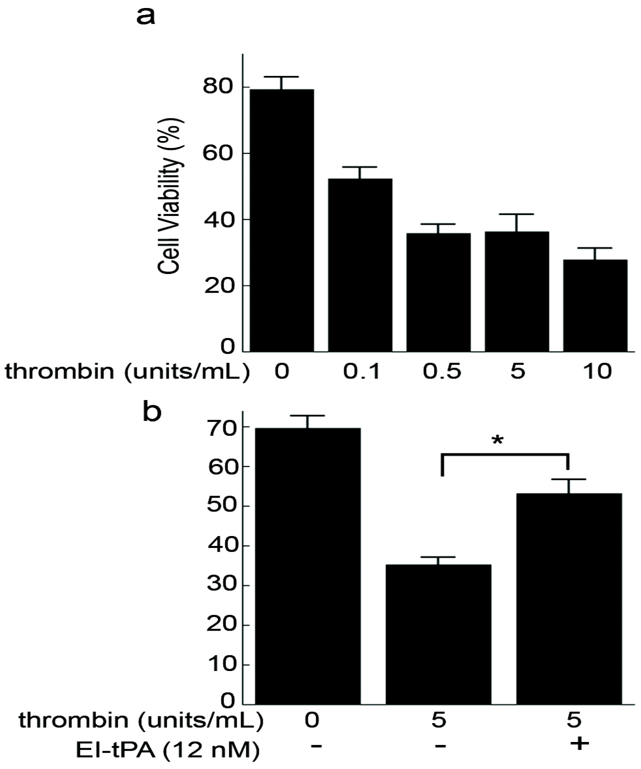

Supplementary Figure S1 (related to Figure 1h). Effects of thrombin on *hi*NPC *in vitro.*
(a) Thrombin dose-dependently reduces *hi*NPC viability. (**b**) Thrombin (5 units/mL) induced cell death is reduced by simultaneous treatment with 12 nM EI-tPA (mean±SEM, *n*=3 per group, *P<0.05 by ANOVA and Tukey's *post hoc* test) .

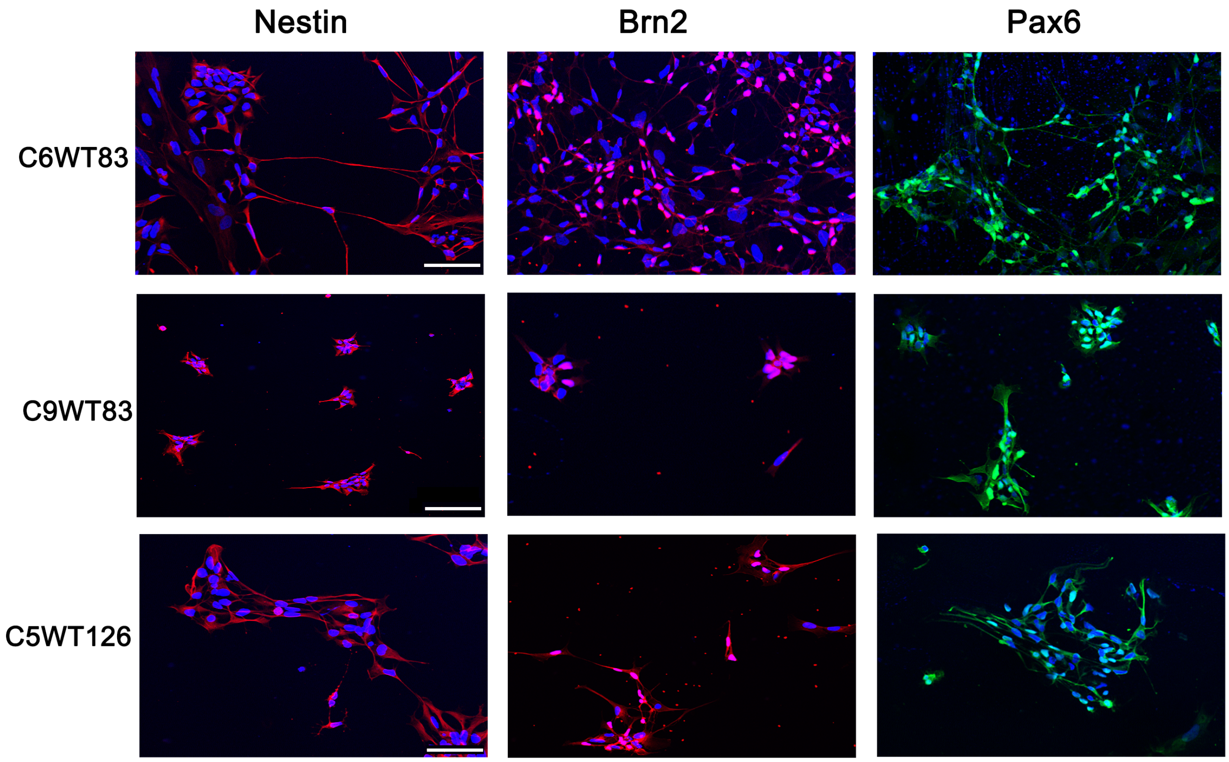

Supplementary Figure S2. Immunofluorescence of stem cell/neural progenitor cell biomarkers. Representative IF microscopy images of three distinct iPSC derived *hi*NPC lines (C6WT83, C9WT83, C5WT126) from two separate healthy patients. Cultured *hi*NPC are immunostained to detect Nestin (red), Brn2 (red) and Pax6 (green). Nuclei are labeled with Dapi (blue) (*n*=3-4 per group). Scale bar 100 μm.

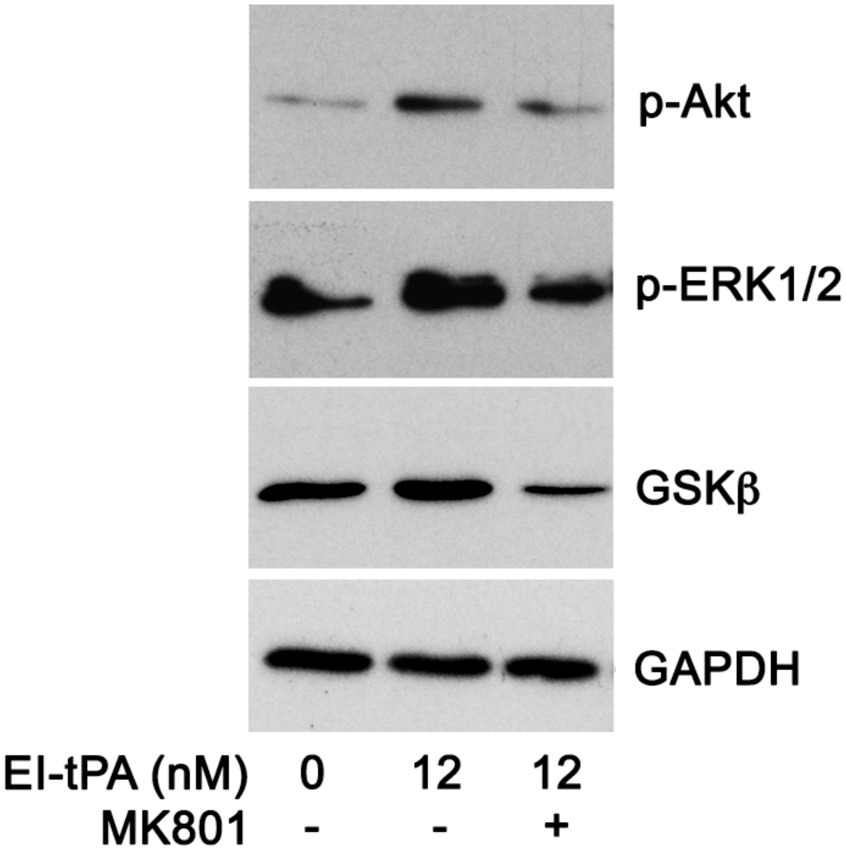

Supplementary Figure S3. EI-tPA induced activation of Akt, GSKβ and ERK1/2 pathways in a NMDA-R dependent manner in a second clone, C5WT126. *hi*NPCs were treated with EI-tPA (12 nM) for 15 min. In some wells, MK801 (1 μM) was added prior to EI-tPA. Equal amounts of cellular protein (10 μg) were subjected to immunoblot analysis to detect phospho-Akt, and reprobed to detect phospho-ERK1/2, phospho-GSKβ and GAPDH. Images represent (n=2-3 independent studies).

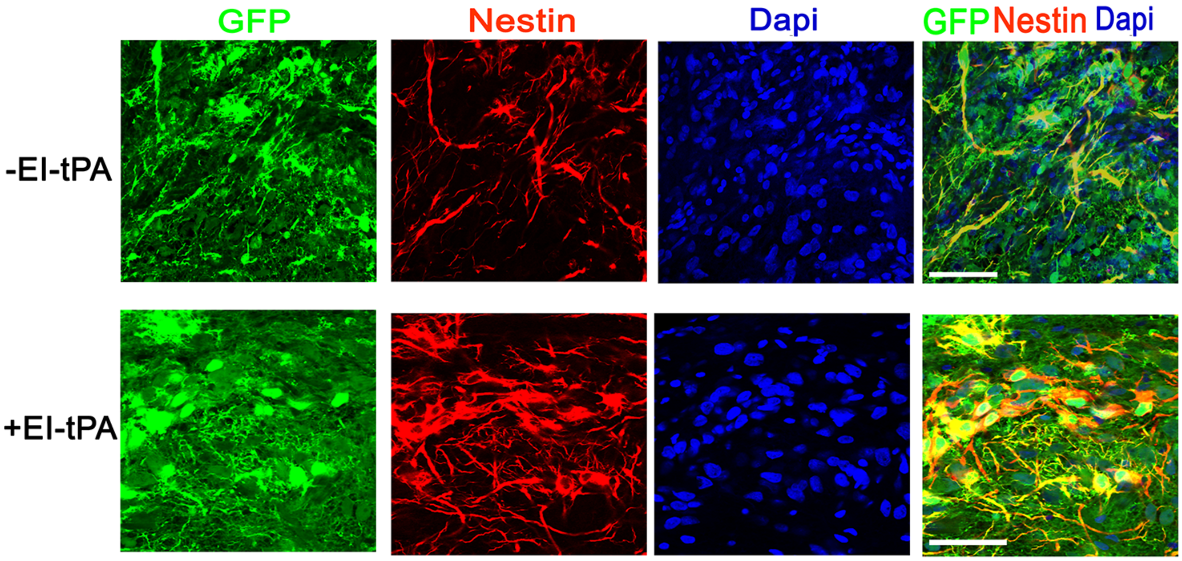

Supplementary Figure S4. Immunofluorescence of GFP and Nestin at the lesion site 8 weeks after SCI. Single panel resolution of *hi*NPC (green), the stem cell marker, Nestin (red), nuclei labeled with Dapi (blue) showing co-localization (combined) in grafted *hi*NPC*.* Scale bars for images (400x) are 50 μm.

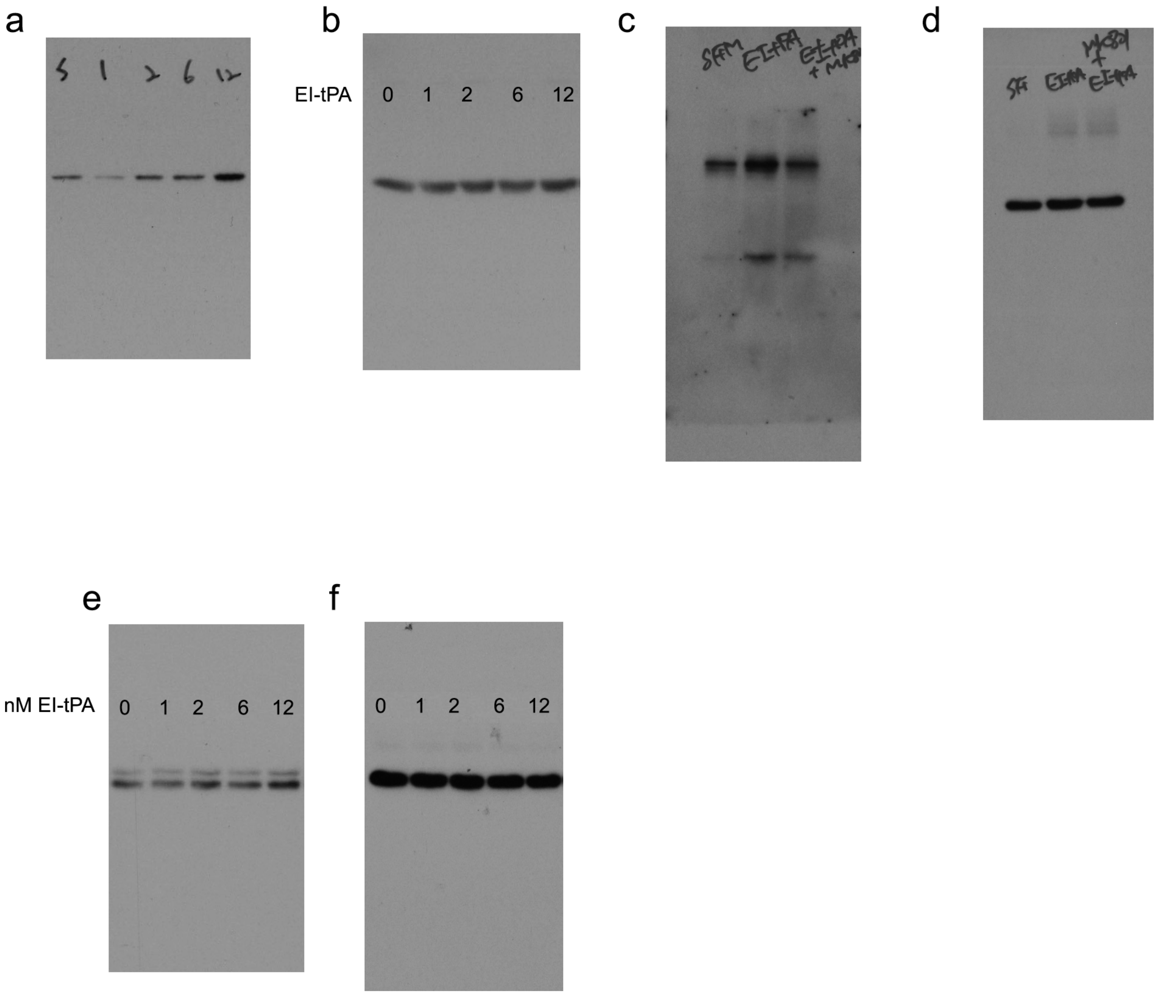

Supplementary Figure S5. Full length blots presented in the manuscript (Figure 1). (**a**) pAkt (60 kDa) in Fig. 1c; (**b**) same pAkt blot stripped and immunoblotted with GAPDH (37 kDa) for load control; (**c**) pAkt in Fig. 1c; (**d**) same pAkt blot stripped and immunoblotted with GAPDH for load control; (**e**) pERK1/2 (42-44 kDa) in Fig. 1e; (**f**) same pERK1/2 blot stripped and immunoblotted with GAPDH.

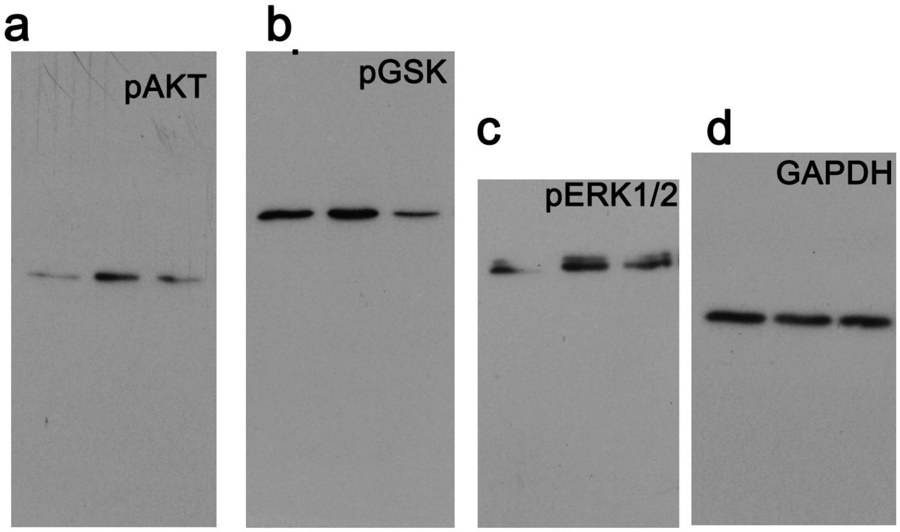

Supplementary Figure S6. Full length blots presented in the Supplementary Data (Figure S3). (**a**) pAkt (60 kDa) (**b**) same pAkt blot stripped and immunoblotted with pGSKβ (46 kDa), (**c**) pERK1/2 (42-44 kDa) and lastly (**d**) GAPDH (37 kDa) load control.

ENSEMBL	ENTREZID	SYMBOL	logFC
ENSRNOG00000029264	54261	Kcnn1	0.5681789
ENSRNOG00000009596	103689945	LOC103689945	0.4220871
ENSRNOG00000026415	314981	Col14a1	0.3895860
ENSRNOG00000018471	100362339	LOC100362339	1.3047170
ENSRNOG00000029792	291015	Ogn	1.3122639
ENSRNOG00000024899	498335	Cxcl13	1.5599194
ENSRNOG00000002501	364073	Ddx3y	1.6609666
ENSRNOG00000024382	304966	Fcgr3a	1.8320943
ENSRNOG00000018808	117064	Vip	2.0345267
ENSRNOG00000056612	286931	Unc13c	2.3767155
ENSRNOG00000046904	293667	B4gat1	5.9183165

Table. S1. Most differentially expressed genes in DRGs isolate from T3SCI rats grafted with hiNPC or not. Log Fc is shown for hiNPC grafted/not grafted.

Movie. S1. Representative video demonstrating complete hindlimb paralysis in rats 15 weeks after being subjected to T3SCI.

Movie. S2. Representative video demonstrating locomotor activity in rats subjected to T3SCI and then treated with *hi*NPCs. The movie was collected 15 weeks after T3SCI.

Movie. S3. Representative video demonstrating locomotor activity in rats subjected to T3SCI and then treated with EI-tPA-treated *hi*NPCs. The movie was collected 15 weeks after T3SCI.

Supplementary Methods

Human Induced (*hi*) Neural Progenitor Cells (NPC)

Fibroblasts were cultured in Minimum Essential Medium (Invitrogen) supplemented with 10% fetal bovine serum (HyClone Laboratories). Retrovirus vectors containing the Oct4, c-Myc, Klf4 and Sox2 human cDNAs from were obtained from Addgene. Two days after infection, human fibroblasts expressing these vectors were plated on a monolayer of mitotically inactivated mouse embryonic fibroblasts (Chemicon) in hESC medium. After 2 weeks, iPSC colonies were directly transferred to Matrigel-coated dishes (BD) in the absence of feeder cells in mTeSR™ medium (StemCell Technologies). To generate *hi*NPC*s*, iPSCs maintained in mTeSR™ medium were switched to N2 medium (DMEM/F12 supplemented with 1X N2 NeuroPlex Serum-Free Supplement (Gemini) with the dual SMAD inhibitors, 1 µM of dorsomorphin (Tocris Bioscience) and 10 µM of SB431542 (Stemgent) for 48 h. After two days, colonies were scraped off and cultured under agitation (95 rpm) as embryoid bodies (EB) for seven days using N2 media with dorsomorphin and SB431542. Media was changed every other day. EBs were then plated on Matrigel™-coated dishes, and maintained in DMEM/F12 supplemented with 0.5X of N2 supplement, 0.5X Gem21 NeuroPlex Serum-Free Supplement (Gemini), 20ng/mL basic fibroblast growth factor (bFGF, LifeTechnologies) and 1% penicillin/streptomycin (P/S). After 7 days in culture, formed rosettes from the plated EBs were manually selected, gently dissociated with StemPro Accutase (LifeTechnologies) and plated onto 10 µg/mL poly-L-ornithine (Sigma) and 5 µg/mL laminin (LifeTechnologies) coated plates. *hi*NPCs were maintained in DMEM/F12 with N2, Gem21, bFGF and P/S. *hi*NPCs were immunopositive for Nestin and were uncommitted to a neuronal or glial lineage prior to transplantation. To visualize the grafted cells *in vivo*, *hi*NPC lines expressing EGFP under the control of the ubiquitin (cell type non-specific) promoter were prepared by infection with a

lentiviral vector (HIV1-UBQ-EGFP; 10 M.O.I.). All the cell lines tested negative for mycoplasma contamination.

Immunoblot Analysis

Cellular extracts of *hi*NPCs were prepared in RIPA buffer. The protein content of each extract was determined by bicinchoninic acid (BCA) assay. An equivalent amount of protein was subjected to 10% SDS-PAGE and electro-transferred to nitrocellulose membranes. Blots were blocked with 5% nonfat dry milk and subsequently incubated with primary polyclonal antibodies to phosphorylated (p) ERK1/2 (1:1000; Cell Signaling; 9101S), pAkt (1:1000; Cell Signaling; 4060), pGSKβ (1:1000; Cell Signaling; 9336) or GAPDH (1:5000; Sigma Aldrich ABS16) in 0.1% BSA in Tris buffered saline with tween-20 (TBST) overnight at 4° C. Antibody-binding was detected by HRP-conjugated species-specific secondary antibodies (1:5000; Cell Signaling) and enhanced chemiluminescence (GE Healthcare). Blots were scanned (Canoscan) and densitometry was performed using Image J software and confirmed using an Azure c600 Gel Imaging system. For re-probing, blots are stripped with Restore (Thermo Fischer, 46430) for 15 minutes at RT. Blots are then washed in TBST for 15 minutes.

Trypan Blue Exclusion Studies

Thrombin (0-25 units/mL) was added to *hi*NPCs at a plating density of 200,000 cells/well in 6-well plates for 18 h. In some cases, EI-tPA was added simultaneously with thrombin (5 units/mL). Cells were scraped off culture flasks coated with 10 µg/mL poly-L-ornithine (Sigma) and 5 µg/mL laminin (LifeTechnologies) and re-suspended. 20 µL of this suspension was mixed with 20 µL trypan blue and counted in a hemocytometer. Five grids were counted per treatment. Data are expressed as mean ± SEM

Motor and Sensory Testing

The BBB open field 21 point locomotion rating was assessed by the established protocol of Basso *et al*. (33) which allows for scoring specific joint movements. Pain-related behavior testing was conducted in plexiglass chambers placed on a smooth stainless steel grid platform, allowing access from underneath the rat. The 50% paw withdrawal threshold (PWT) to a series of calibrated von Frey filaments (Kom Kare, Middletown, OH, USA) was assessed in the center of paw before surgery (baseline) and at regular intervals following SCI using the up-down method (62). Pain behavior testing of both hindpaws and forepaws was determined as previously described (27). All scoring and testing were conducted prior to surgery and weekly beginning one week after T3SCI. The set of filaments was calibrated to exert a force of 0.2-15 g. Values obtained from each pair of forepaws or hindpaws were averaged per time point. All motor and sensory experiments were conducted by investigators who were blinded to the treatment groups.

Cell Fate Mapping by Immunofluorescence

For *hi*NPCs *in vitro*, cells are fixed in 4% PFA for 15 min. Cells are permeabilized (0.3% Triton X100) and blocked in horse serum. Primary antibodies, Pax6 (GeneTex, 1:100), Brn2 (Genetex; 1:500) and Nestin (R&D Systems; 1:500; MAB1259) were used and subsequently incubated in Alexa-Fluor 488 and/or Alexa-Fluor 594 and/or Alexa-Fluor 647 conjugated anti-goat or anti-donkey secondary antibodies (Thermo Fisher A11055, A11058, SA5-10062, SA5-10064, 1:500) for 1 h and DAPI (Sigma D9542, 0.5 μg/μl, to label nuclei) for 5 min. In control studies, the primary antibody was omitted.

Tissue from the graft site and T6-T8 was collected from deeply anesthetized rats that were transcardially perfused with 4% paraformaldehyde (PFA). The spinal columns were removed

and kept in 4% PFA overnight at 4 °C and transferred to 30% sucrose prior to dissection and sectioning. Spinal cord dura was removed and cut in the transverse plane into 1.0- or 1.5-cm-long blocks as previously described (27). The block containing the lesion was cut into 30-μm-thick horizontal sections using a cryostat. Additional blocks of tissue were cut into 20-μm-thick transverse sections. Tissue sections were stored at –20°C in tissue cryoprotectant solution (TCS; 25% glycerin (vol/vol) and 30% ethylene glycol (vol/vol) in 0.5 M phosphate buffer). Tissue sections were blocked in 5% TNB and incubated overnight at 4° C with primary antibody directed against GFAP (1:1000; EMD Millipore mab360 or 1:1500 Encor Bio no. CPCA-GFAP); GFP (rabbit, Thermo Fisher no. 6455, 1:1,500); hNu (MAB1281, clone 235-1; EMD Millipore; 1:200); class III β-tubulin (mouse, Promega G-7121, 1:1,000); choline acetyltransferase (ChAT) (AB144P; EMD Millipore; 1:200); and MNX1/HB9 (ABN174; Sigma Aldrich). Sections were washed and then incubated in Alexa-Fluor 488 and/or Alexa-Fluor 594 and/or Alexa-Fluor 647 conjugated anti-goat or anti-donkey secondary antibodies (Thermo Fisher A11055, A11058, SA5-10062, SA5-10064, 1:500) for 1 h and DAPI (Sigma D9542, 0.5 μg/μl, to label nuclei) for 5 min. In control studies, the primary antibody was omitted.

Slides of tissues or cells are visualized using an Olympus FluoView FV1000 confocal microscope at 10x to 60x magnification. Up for three channels were to detect each fluorophore's excitation and emission spectrum. Single plane images were taken at a scanning speed of 10 μs/pixel. The exposure time was set based on control tissue with no secondary fluorophore. Quantification of hNu^+ nuclear size and number was performed using Image J and Imaris software. The number of GFP-labeled human profiles in the spinal cord was counted in central, ventral and dorsal regions. Quantification by Image J of 2 coronal sections from three animals was included.

References

62. Chaplan, S. R., Bach, F. W., Pogrel, J. W., Chung, J. M. & Yaksh, T. L. Quantitative assessment of tactile allodynia in the rat paw. *J. Neurosci. Meth.* **53**, 55–63 (1994).
